# Relapsed Wilms’ tumor in pediatric patients: challenges in low- to middle-income countries—a single-center experience

**DOI:** 10.1186/s43046-020-00032-6

**Published:** 2020-05-01

**Authors:** Wael Zekri, Dalia M. Yacoub, Asmaa Ibrahim, Youssef Madney

**Affiliations:** 1 Department of Pediatric Oncology, National Cancer Institute, Cairo University, Fom El-khalig Square, Kasr El-Aini St, Cairo, 11796 Egypt; 2 Department of Pathology, National Cancer Institute, Cairo University, Fom El-khalig Square, Kasr El-Aini St., Cairo, 11796 Egypt

**Keywords:** Wilms’ Tumor, Relapse, Radiotherapy, Risk stratification, Post-relapse survival

## Abstract

**Background:**

Wilms’ tumor (WT) affects one in 10,000 children and accounts for 5% of all childhood cancers. Although the overall relapse rate for children with WT has decreased to less than 15 %, the overall survival for patients with recurrent disease remains poor at approximately 50 %. The aim of the study to evaluate the outcome of relapsed Wilms’ tumor pediatric patients treated at the National Cancer Institute (NCI), Egypt, between January 2008 and December 2015.

**Results:**

One hundred thirty (130) patients diagnosed with WT during the study period, thirty (23%) patients had relapsed. The median follow up period was 22.3 months (range 3.6–140 months). The Overall Survival (OS) was 30.9% while the event-free survival (EFS) was 29.8% at a 5-year follow up period. Median time from diagnosis to relapse was 14.4 months. A second complete remission was attained in 18/30 patients (60%). The outcome of the 30 patients; 11 are alive and 19 had died. Three factors in our univariate analysis were prognostically significant for survival after relapse. The first was radiotherapy given after relapse (*p* = 0.012). The 5-year EFS and OS for the group that received radiotherapy were 41.9% versus 16.7% and 11.1% respectively for those that did not. The second was the state of lymph nodes among patients with local stage III (*p* = 0.004). Lastly, when risk stratification has been applied retrospectively on our study group, it proved to be statistically significant (*p* = 0.029).

**Conclusion:**

Among relapsed pediatric WT, radiotherapy improved survival at the time of relapse and local stage III with positive lymph nodes had the worst survival among other stage III patients.

## Background

Wilms’ tumor affects one in 10,000 children and accounts for 5% of all childhood cancers. More than 80% of children are diagnosed with Wilms’ tumor below the age of five years, and the median age at diagnosis is 3.5 years [1]. The outlook for children with newly diagnosed Wilms’ tumors (WT) has improved dramatically with the advent of multimodal therapy, which includes surgery, chemotherapy, and for some, radiation therapy, with survival rates currently approaching 90 % [2]. Although the overall relapse rate for children with WT has decreased to less than 15 %, the overall survival for patients with recurrent disease remains poor at approximately 50% [3, 4].

Long-term follow-ups are mandatory, as approximately 15% of patients with favorable- histology WT and 50% of patients with anaplastic WT had recurrences. Most recurrences occur within two years of diagnosis, although the recurrences have been known to occur even after 25 years of initial treatment [5]. Before the 1990s, in many relapse WT patients, the same chemotherapy agents were generally used for the treatment of both primary and recurrent disease. The salvage rate for patients with recurrent favorable- histology WT used to be 25–40% [6]. Outcomes started improving up to 60% in the last 15–20 years when modern treatment combinations were tried [7].

Due to the small numbers of relapsed patients, advancements in the treatment of these patients have remained a challenge. Investigators from different cooperative groups have evaluated the role of different therapeutic strategies in an attempt to improve the outcomes of relapse WT. Therapy for these patients depends on the characteristics of their primary disease, the extent of previous therapy, and time from the initial diagnosis to relapse. The general principle of management shifted to include drugs that are not used during primary chemotherapy, using a risk-stratified approach. Patients with relapsed tumors are classified into three groups in the UMBRELLA protocol, group AA, group BB, and group CC, based on initial histology and the first line of treatment used [8].

Our study aimed at estimating the number of relapsed Wilms’ tumor patients between January 2008 and December 2015. Also, to correlate different factors in patients’ demographics, initial disease data, relapse data, post-relapse survival outcome, and defining factors related to the outcome.

## Methods

This is a retrospective study on pediatric patients of relapse WT from January 2008 till December 2015. Patients’ medical records were reviewed for different data regarding the initial disease phase and at the relapse phase also for patients’ demographics. The initial disease stage was assigned the Children Oncology Group (COG) clinicopathologic staging of WT. Reference Pathology reports were reviewed for pathological subtype, local tumor stage, resection margins, renal capsule, and lymph node involvement. Information about first-line therapy and the response was collected. Operative details, lymph node dissection, tumor spillage, capsule rupture and/or perioperative complications. Histopathology for documentation of relapse was reviewed. Radiology reports were reviewed for the site of relapse. Post-relapse chemo-radiotherapy and surgery were analyzed in detail.

### Initial treatment protocol of the entire study group

Most studied patients were treated initially according to a modified, COG-based protocol (i.e., no LOH of 1p and 16q as a risk factor and without an upfront surgical approach in all cases). Stage I and II FH tumors received 18 weeks EE4A [vincristine (VCR) and dactinomycin (AMD)] without radiotherapy (RTH). Stage III received 24 weeks of DD4A [VCR, AMD, and DOX (doxorubicin)] together with local radiotherapy. Stage IV patients were treated locally with surgery and RTH as either stage I/II or III FH WT. Regimen M (cyclophosphamide and etoposide alternating with vincristine, doxorubicin, and dactinomycin for a total of 33 weeks) with radiotherapy for stage 4 patients with pulmonary metastasis with a slow response after 6w DD4A regimen or extrapulmonary metastatic disease. For the unfavorable histology (UH) group, stage I, II, and III, focal anaplasia, together with stage I diffuse anaplasia, received DD-4A regimen. Stage IV, focal anaplastic were treated with the UH regimen (cyclophosphamide, carboplatin, and etoposide alternating with vincristine, doxorubicin, and cyclophosphamide for 30 weeks) and RT to the metastatic sites. Patients with stages II, III, and IV diffuse anaplastic received regimen UH and RT (Additional file 1).

### Risk stratification of relapse WT

Retrospectively, we applied the umbrella protocol risk stratification i.e. groups AA, BB, and CC [8]. *Standard Risk*: defined as patients with favorable histology WT with relapse after therapy with only vincristine and/or actinomycin D. Those were found. *High Risk*: defined as patients with favorable histology WT with relapse after therapy with three or more agents (at least including doxorubicin). *Very High Risk*: defined as patients with recurrent anaplastic—regardless of adoption of primary chemotherapy or primary surgery- or post-chemotherapy blastemal-type WT.

### Relapsing Wilms’ tumor regimen treatment protocols

All patients received chemotherapy for their first relapse. The most commonly used regimens were ICE and CCE for six cycles on average. The relapsing protocol has also been used for 2 cases in our study; this regimen consists of alternating two courses of the carboplatin–etoposide and one course of cyclophosphamide every 3 weeks for 25 weeks. Patients were evaluated for response assessment after every two cycles (Additional file 1: Table S1).

Radiotherapy for relapsed patients was given for most of relapsed Wilms’ tumor patients, Radiation delivery mainly using 3D conformal radiotherapy (3DCRT), Treatment modality using photon beam with energy ranging from 6 to 10 MV according to patient’s separation (i.e. thickness) but in most cases 6 MV was sufficient, Children with abdominal recurrence received 1980 cGy, the dose for lung irradiation, using 150 cGy daily fractions, generally reaches 1200 cGy. Patients with non-respectable liver nodules usually received up to 1980 cGy if the entire liver was diffusely involved. Patients who achieved a complete remission following chemotherapy and/or hepatic resection did not receive radiation therapy to the liver.

### Statistical analysis

Survival analysis was done using the Kaplan-Meier method and a comparison between two survival curves was done using a log-rank test. All tests were two-tailed. Overall survival (OS) was calculated as the period between the date of the first relapse and the date of death from any cause or the date of the last follow up. Event-free survival (EFS) was calculated as the period between the time of the first relapse and the date of disease progression or second relapse, date of last follow up or date of death. *p* value of ≤ 0.05 was considered statistically significant.

### Results

Among the 130 patients diagnosed with Wilms’ tumor between 2008 and 2015, 39 patients (30%) had relapsed. Nine patients were excluded for incomplete data or due to receiving treatment outside. In the remaining 30, 18 were males (60 %) 12 females (40%). The median age at diagnosis was four years. No congenital anomalies were documented. Tumors located in the right kidney were found in 12 patients (40%) and those in the left kidney were in 16 patients (53%). One patient had bilateral with pulmonary metastasis and another one had an extrarenal (retroperitoneal) WT.

Initial stages I, II, III, and IV was documented in 7(23.3%), 3(10%),12(40%), and 7 cases (23.3%) retrospectively. Only one patient had bilateral WT (3.3%). Initial metastasis was documented in 8/30 patients (26.6%). Lung metastases were found in all metastatic cases and combined liver and lung metastases were only found in 2/8 cases. All stage IV cases had local stage III, which made a total of 19 cases (63.3%). Favorable histology (FH) was documented in 24 patients (80%) and unfavorable histology (UH) in 6 patients (20%). Initial staging and treatment regimen are summarized in (Table 1)

**Table 1:** Initial management of the entire study group

ID	Pathology*	Initial metastatic status	Local staging	Post-operative CTH regimen	Received RTH
1	FH	NO	III	EE4A	No
2	FH	NO	III	WTS 1 **	Yes
3	UFH	NO	III	Regimen M	Yes
4	UFH	Lung	III	DD4A	Yes
5	FH	NO	II	EE4A	No
6	FH	NO	I	No	No
7	FH	lung	III	Regimen M	Yes
8	FH	Lung and liver	III	Regimen M	Yes***
9	FH	NO	III	EE4A	No
10	FH	NO	III	EE4A	No
11	UFH	NO	I	NA	No
12	FH	Lung	II	DD4A	Yes
13	FH	NO	II	NO	No
14	FH	Lung	III	DD4A	Yes***
15	FH	NO	III	DD4A /Vp16 Cyclo/Carbo	Yes
16	FH	NO	III	DD4A	Yes
17	UFH	NO	III	DD4A	Yes
18	UFH	NO	I	EE4A	No
19	FH	NO	III	DD4A/Regimen M	Yes
20	FH	NO	III	VCR/COS	Yes***
21	FH	Lung	III	WTS 1 2000*	Yes
22	FH	NO	I	VCR/COS/ VCR/ADR	No
23	FH	NO	I	EE4A	No
24	FH	NO	III	DD4A	Yes
25	FH	NO	II	DD4A	No
26	FH	NO	I	EE4A	No
27	UFH	NO	III	DD4A	Yes
28	FH	Lung and liver	III	DD4A	Yes
29	FH	NO	I	EE4A	No
30	UFH	Lung	III	DD4A	Yes

* FH favorable histology, *UFH* unfavorable histology, *RT* radiotherapy, *WLI* whole lung irradiation, *VCR* vincristine, *COS* Cosmogen, *ADR* adriamycin, *Cyclo* cyclophosphamide, *Carbo* carboplatin, *ETO* etoposide

**WTS 1 2000: VCR 1.5 mg/m2 COS 45mcg/kg DOX 40 mg/m2 CYCLO 1200 mg/m2

***Whole abdominal irradiation was given to patients 8 (IVC involvement), 14 (extensive surgery), and 20 (spillage). EE4A: consists of VCR + COS, DD4A: VCR + COS + ADR, REG M: VCR + COS + ADR + CYCLO+ETO

Median time from diagnosis to relapse was 14.4 months; early relapse (less than 12 months) in 43.3 % (*n* = 13) while 56.7% (*n* = 17) relapsed after 12 months. Local relapse was found in 11/30 cases (36.3%). Distant relapse was in 10/30 patients (33.3%), all 10 patients had lung relapse and two of which were associated with liver or bone. Combined relapse was in 9/30 patients (30%).

Diagnosis of relapse was mainly based on radiological confirmation of recurrence; however pathological confirmation of relapse was confirmed in 9/30 (30%) patients. Anaplasia was documented in 2 cases only. Blastemal predominance was present in two patients. None of the pathology documented at the time of relapse was similar to the pathology at initial disease presentation except for one patient that had diffuse anaplasia initially and at relapse. The other patient with anaplastic Wilms’ tumor at relapse showed initially favorable histology. Despite the role of molecular genetics at diagnosis and at relapse like tumor-specific loss of heterozygosity (LOH) for chromosomes 1p and 16q but not done as unavailability in our center.

During the first-line treatment: Upfront nephrectomy was performed in 21/30 patients (70%). Intraoperative capsule rupture was documented in 2/30 cases (6.6%), whereas tumor spillage in 1/30 cases (3.3%). Lymph node sampling proved pathological involvement in 5/30 cases (16.7%). Neoadjuvant chemotherapy was given to 9/30 patients (30%); radiotherapy was given to 17/30 patients (56.7%), while anthracycline in 18/30 patients (60%).

After relapse, surgical excision of the relapsed tumor was feasible in 17 patients (56.7%). Radiotherapy was a major part in the treatment of relapsed cases, eighteen (18) patients (60%) had received radiation to sites of relapse. Flank irradiation 1080 cGy was given to six patients with local recurrence and whole abdominal radiotherapy 1980 cGy was given to six patients with a boost to para-aortic lymph nodes in two patients. Five patients received lung irradiation 1200 cGy. Two patients with non-respectable liver nodules received up to 1980 cGy and one patient received radiotherapy 3060 cGy to metastatic D12–L1 vertebrae.

All patients received chemotherapy for their first relapse. The most commonly used regimens were ICE and CCE for six cycles on average. The relapsing protocol has also been used for 2 cases in our study; this regimen consists of alternating two courses of the carboplatin–etoposide and one course of cyclophosphamide every 3 weeks for 25 weeks. Patients were evaluated for response assessment after every two cycles.

A second complete remission (CR) was attained in 18 patients (60%) and partial remission (PR) in 3 patients (10%). One patient (3.3%) showed a stationary response (SD) and 7 patients (23.3%) had progressive disease (PD). Out of the 18 patients that achieved a second CR, 8 continued to follow up in CR, while 8 had a second relapse, one patient lost follow-up, and one patient died on treatment. In the patients with the second relapse, only 3 received a third treatment line. One is alive under therapy while the remaining had died. The final outcome of the 30 patients: 11 are alive and 19 dead (Table 2).

**Table 2:** Description of the whole study group regarding different prognostic variables and survival

I.D number	Stage	Time of relapse	Relapse site	Treatment at relapse				Status	Risk classification	Alive/dead
				CTH	No of cycles	Surgery	RT			
1	III	Early	Local, liver	ICE	6	None	Yes	2nd CR	SR	A
2	III	Early	CL kidney	ICE	8	None	No	2nd CR	HR	A
3	III	Late	Liver, lung	ICE	8	None	Yes	PD	VHR	D
4	IV	Late	Lung	CCE	6	None	Yes	2nd CR	VHR	A
5	II	Early	Local, lung	Relapsing protocol	25 wks	None	Yes	2nd CR	SR	A
6	I	Early	Local, mediastinal	ICE	9	Excision	Yes	RL2	SR	A
7	IV	Early	Lung	ICE	6	Metastatectomy	No	TRM	HR	D
8	IV	Late	Lung	ICE	6	Metastatectomy	No	2nd CR	HR	A
9	III	Late	Lung, bone	Relapsing protocol	25 wks	None	Yes	2nd CR	SR	A
10	III	Late	CL kidney	VAD then DD4A	6 + 25 wks	Right partial nephrectomy	No	RL2	SR	D
11	I	Early	Local	CCE	6	Excision	Yes	TRM	VHR	D
12	V	Late	Left renal mass	CCE	3	Lt radical nephrectomy	Yes	TRM	HR	D
13	II	Late	Lung	DD4A	22 wks	None	No	PD	SR	D
14	IV	Late	Local	CCE	4	None	No	PD	HR	D
15	III	Late	Paraaortic LN	DD4A*	2 + 7	Excision	Yes	2nd CR	HR	A
16	III	Late	Lung, mediastinal	CCE	4	None	No	TRM	HR	D
17	III	Late	Paraaortic LN	CCE	6	Excision	Yes	PD	VHR	D
18	I	Late	Lung	DD4A	25 wks	Metastatectomy	Yes	2nd CR	VHR	A
19	III	Early	Lung, pelvic LN	ICE	3	None	No	TRM	HR	D
20	III	Late	Local, paraaortic LN	CCE	8	Excision	Yes	RL2	HR	D
21	IV	Late	Lung	ICE	8	Metastatectomy	No	RL2	HR	D
22	I	Late	Local	ICE	6	Exploration	Yes	RL2	HR	D
23	I	Early	Lung, pelvic LN	CCE	6	Excision	Yes	2nd CR	SR	A
24	III	Early	Local, liver, abdominal LN	ICE	8	None	Yes	RL2	HR	D
25	II	Early	Abdominal LN, mediastinal	CCE	4	WLE	No	TRM	HR	D
26	I	Early	Paraaortic LN, abdominal pelvic LN	UH	31 wks	Excision	Yes	2nd CR	SR	A
27	III	Early	Lung	CCE	6	None	No	PD	8.00	D
28	IV	Late	Abdominal LN	ICE	6	None	Yes	RL2	HR	D
29	I	Late	Local, lung, abdominal LN	ICE	7	Exploration	Yes	PD	SR	D
30	IV	Early	Lung	CCE	6	Metastatectomy	No	RL2	HR	D

FH favorable histology, *UFH* unfavorable histology, *Early relapse* < 12 months, *Late relapse* > 12 months *LN* lymph nodes, *CL* contralateral kidney, *Wks* weeks, *A* alive, *D* dead, *RL2* second relapse, *PD* progressive disease, *CR* complete remission, *TRM* treatment-related mortality, *SR* standard risk, *HR* high risk, *VHR* very high risk

Several adverse prognostic factors were recorded in our study; unfavorable histology at diagnosis, initial local stage III, recurrence < 12 months, abdominal and lung relapse, previous anthracycline therapy, and time from nephrectomy < 12 months. The patients having just one bad prognostic factor were 23.3% (*n* = 7), similarly were the patients having 2 or 3 adverse prognostic factors 23.3% each. Those with more than 3 formed collectively 26.7% (*n* = 8). One patient had none of the previously mentioned bad prognostic factors.

**Fig. 1:**
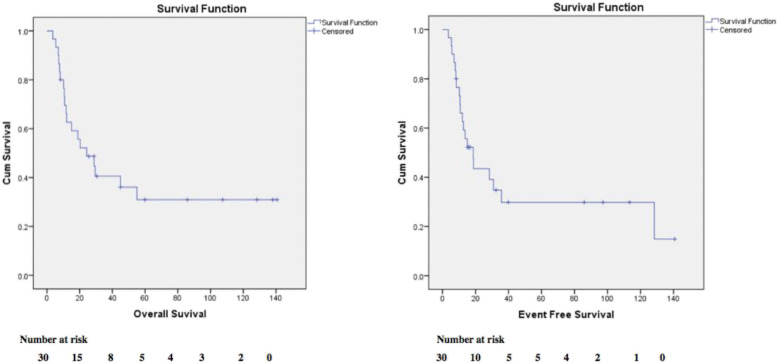
Survival outcome for study group patients

The median follow up period was 22.3 months (ranged from 3.6 to 140.7 months). The 5-year (OS) was 30.9% while the 5-year (EFS) was 29.8% (Fig. 1). Median time from diagnosis to relapse was 14.4 months. The 5 year OS for relapse < 12 months from diagnosis was 44.8% while that for relapses > 12 months was 18.4% and that was not statistically significant (*p* value = 0.478). Favorable histology had better survival rates than those with unfavorable histology but it was also statistically insignificant. The local stage did affect OS but was not statistically significant. The 5 year OS for stages I, II, and III was 47.6%, 25%, and 24.2% respectively.

**Fig. 2:**
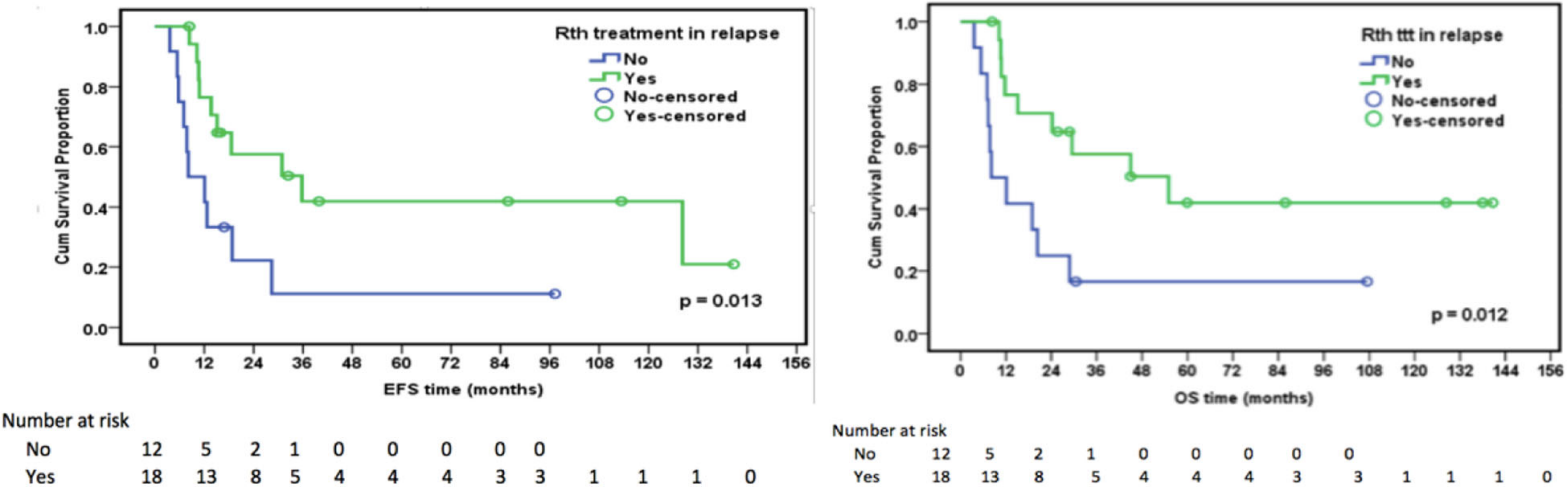
Impact of radiotherapy on survival outcome among relapsed Wilms’ tumor patients

**Fig. 3:**
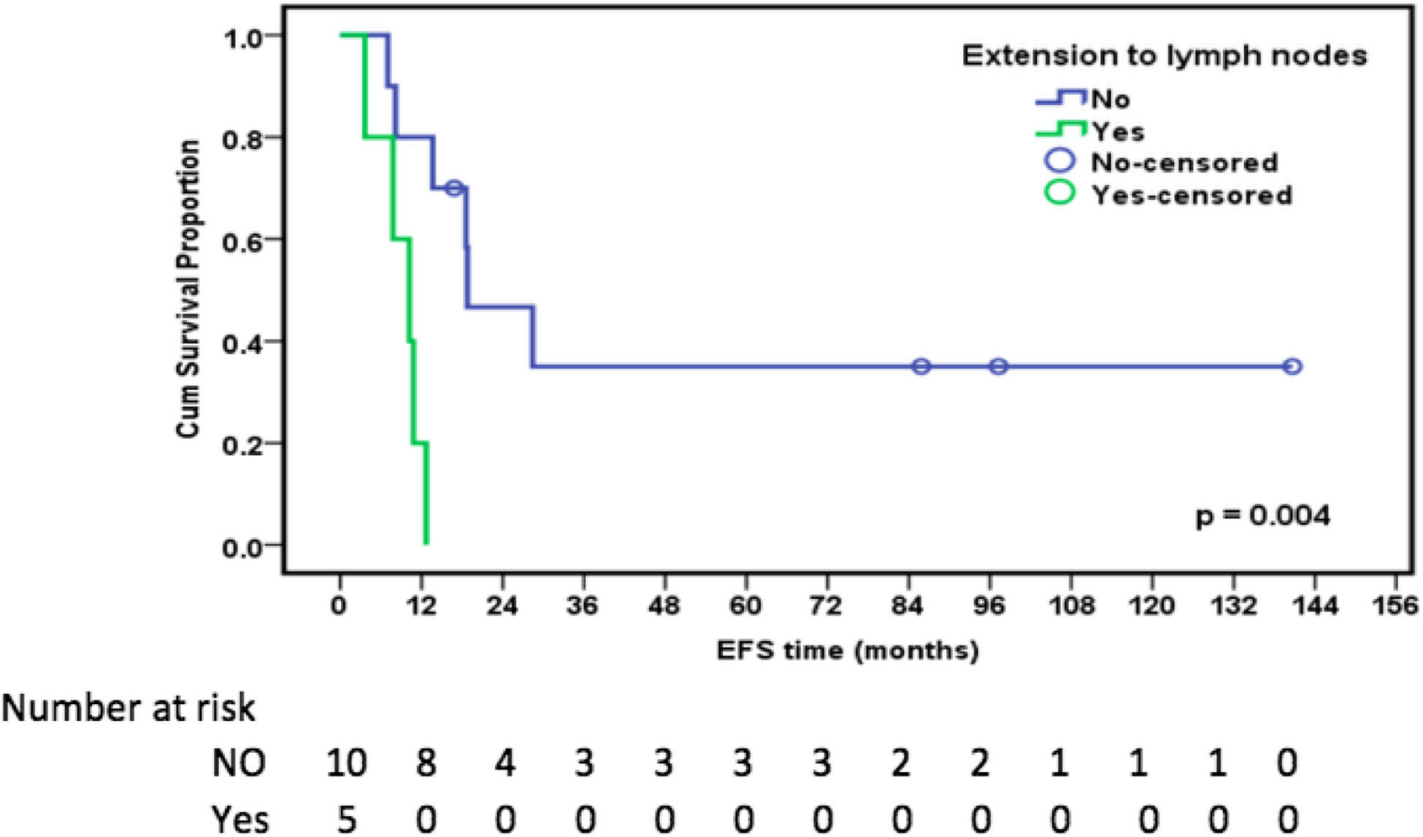
Impact of lymph node extension on EFS among relapse stage III Wilms’ tumor patients

The prognostically significant factors for survival were radiotherapy given after relapse with The 5 year OS for the group that received radiotherapy was 41.9% versus 11.1% for those who did not (*p* value = 0.012) (Fig. 2). The state of lymph nodes in locally stage III was statistically significant, the 5 year EFS for patients with initially negative lymph nodes was 35%, while those with positive lymph nodes (*n* = 15) all died before the end of the first year of follow up, *p* value (0.004) (Fig. 3). Risk stratification has been applied retrospectively in our study group. Standard risk (SR) Group AA, High risk (HR) BB, and Very high risk (VHR) CC formed 33.3%, 46.7%, and 20% of patients respectively. On the correlating risk category with survival, it proved statistically significant (*p* = 0.029).

The 5 year OS for local relapse was 12.5% while that of distant relapse was 40.0%, combined relapse came with the best survival at 44.4%. The affection of survival by the number of adverse prognostic factors was not statistically significant **(**Table 3).

**Table 3:** Impact of different prognostic variables on survival outcome

Variables	No. of patients	Event-free survival at 60 months (5 years)	*P*	Overall survival at 60 months (5 years)	*P*
Total	30	29.8		30.9	
Age					
<5 years	20	38.2	0.091	38.5	0.146
≥5 years	10	NA		NA	
Gender					
Male	18	29.6	0.864	30.0	0.984
Female	12	30.9		34.7	
Interval between diagnosis and relapse					
≤12 months	13	44.9		44.9	0.478
>12 months	17	16.8	0.685	18.4	
Interval between nephrectomy and relapse					
<12 months	13	44.9	0.685	44.9	0.478
> = 12 months	17	16.8		18.4	
No. of adverse prognostic factors					
1 and 2	15	19.3	0.920	21.1	0.821
3,4, and 5	15	38.9		38.9	
Initial metastatic status					
No	22	37.8	0.129	37.8	0.145
Yes	8	NA		NA	
Initial pathology					
Favorable	24	30.3	0.647	31.5	0.681
Unfavorable	6	NA		NA	
Initial systemic stage					
I, II	10	45.0	0.565	45.0	0.511
III	12	28.1		28.1	
IV	8	NA		NA	
Initial local stage					
I	7	47.6%	NA	47.6	NA
II	4	25.0	NA	25.0	
III	19	21.8	NA	24.2	
Site of relapse					
Local	11	12.5	0.934	12.5	0.787
Distant	10	33.3		40.0	
Combined	9	44.4		44.4	
RTH in relapse**					
No	12	11.1	0.013	16.7	0.012
Yes	18	41.9		41.9	
Risk stratification					
Standard	10	57.1	0.029	57.1	0.035
High/very High	20	15		17	
Post-operative residual					
No	24	30.9	0.957	32.3	0.944
Yes	5	25.0		25.0	

** *RTH* radiotherapy

## Discussion

WT is the most common renal tumor of infancy and childhood. Its incidence is one per 10,000 children under the age of 15 years worldwide [1]. Multimodality treatment has resulted in a significant improvement in the 4-year survival, from approximately 30% in the 1930s to more than 85% in the modern era. The overall prognosis in WT remains poor in developing countries [9]. Acquiring information about the cure rate after treatment of relapsed WT is difficult because numbers of patients are small due to the high cure rate after primary treatment. Moreover, interpretation of the published studies is complicated by differences in initial therapy and patient selection [10].

In our study, the relapse rate was high accounting for 30%. The relapse rate reported by other Egyptian studies varied between 17.9% and 24.2% [11].These are all higher than those demonstrated by the 4th NWTS and the 9th SIOP which were 11% and 10% respectively [12]. The median age at initial diagnosis was four years. This was comparable to the study of all relapses in patients registered in the German SIOP/GPOH trials where the median age was 4.5 years [13].

In our study, stage III tumors were the most common (40%), followed by stages I and IV (23.3%) each. Then came stage II (10%) and lastly stage V (3.3%). The stage distribution came slightly different to that in a Swedish study on 13 relapsed cases where stage I formed 38% of cases (*n* = 5), stage II 15% (*n* = 2), stage III 23% (*n* = 3), stage IV 8% (*n* = 1) and stage V 15% (*n* = 2) [14].

In our study, the 5-year post-relapse survival was 30.9% this was approximate to another study in the University of Iowa on 21 patients, where it was reported to be 33% [15]. Likewise, the United Kingdom Children’s Cancer Study Group analyzed 71 children with a 24% post-relapse survival at a median follow-up of 3 years [10]. At the St. Jude Children’s Research Hospital, Williams and colleagues reported a 25% post-relapse survival in 32 patients with a median follow-up of 6.5 years [15]. However, our results were less than those reported by the Koreans where a study conducted in Seoul national university children’s hospital calculated survival to be 40.9% [16]. The outcome of relapses of nephroblastoma in patients registered in the SIOP/GPOH trials and studies, the overall survival after relapse was 48% (median follow-up 5 years) [13].

Radiotherapy given after relapse was a prognostically significant factor for survival (*p* value 0.012). The 5 year EFS and OS for the group that received radiotherapy were 41.9% versus 16.7% and 11.1% respectively for those that didn’t (Table 2). A total of 18 patients have received radiotherapy and 9 are alive from which 7 in second CR. There is limited information available on the role of RTH in the management of relapse WT. In the NWTS report, 30 patients developed solitary pulmonary relapse and they underwent bilateral lung irradiation, and chemotherapy with or without surgical resection; the 4-year post-relapse survivals were 76% and 77%, respectively. Whereas 55 patients had multiple pulmonary nodules at relapse, their 4-year survival after whole-lung RTH and chemotherapy was 44% [17]. NWTS-5 protocol recommends surgical resection of persistent pulmonary nodules after induction chemotherapy, patients with pulmonary relapse who achieve a complete remission after surgical resection receive whole-lung RTH if there is no history of prior pulmonary irradiation [18].

In our study, univariate analysis of proposed risk factors indicated that EFS and OS were significantly associated with individual stage III criteria among patients with local stage III. Of prognostic relevance in this subgroup was the initial state of lymph nodes with a *p* value 0.004. The patients with initially negative lymph nodes had a 5 year EFS of 35%, while those with positive lymph nodes (*n* = 15) all have died before the end of the first year of follow up. This was comparable to another study where each criterion designating stage III was assessed in terms of affecting survival. The EFS and OS were better for patients with negative lymph nodes. The multivariate analysis showed the joined effect of lymph nodes involvement and microscopic residual disease was most predictive of outcome [19]. In another retrospective study of 95 cases over 20 years, 28 cases had a relapse; on multivariate analysis, lymph nodes positivity and anaplasia were independently correlated with disease-specific survival [20].

Reinhard et al. provided an analysis of prognostic factors for post-relapse survival [13]. In their study of 170 relapses of all 1392 children registered in SIOP/GPOH trials from the year 1989 till 2003; it was found that lower disease stage at diagnosis had a better outcome. Stage I and II had a better outcome than stage III (*p* = 0.008). They also found that early relapse within 6 months from diagnosis was a strong predictor of poor survival after relapse (*p* = 0.0001). Furthermore, patients with isolated distant metastasis had a significantly better outcome (OS = 59%) than those with isolated local (OS = 50%) and combined relapses (OS = 12%) (*p* = 0.001). Finally, high-risk histology was one of the adverse prognostic factors for poor post-relapse outcomes [13]. A number of factors were analyzed in our study in relevance to post-relapse survival. Not all factors were of prognostic relevance; however, the small number of our study’s cohort requires further analysis of the proposed factors.

Risk stratification based on umbrella protocol [8] has been applied retrospectively in our study group. Group AA formed 33.3% of patients, Group BB 46.7%, and Group CC formed 20% of cases. On the correlating risk category with survival, it proved statistically significant (*p* = 0.029).

Before the mid-1980s, the OS for relapse WT was 30%. During this era, salvage therapy consisted of vincristine, dactinomycin, doxorubicin, radiation therapy, or surgery. In recent years (Table 4), cyclophosphamide, ifosfamide, cisplatin, carboplatin, and etoposide were shown to be active against relapse WT and multiagent regimens containing these drugs, mostly ICE (ifosfamide, carboplatin, and etoposide) have significantly improved post-relapse survival rates to the 50-60% range [21]. However, the role of high-dose chemotherapy and stem cell rescue (ASCR) in patients with high-risk recurrent WT is not fully defined [24]. A study performed at the Memorial Children hospital in Chicago proved a good outcome after treatment of relapsed Wilms’ tumor with high-dose chemotherapy (HDT) followed by ASCR. Seven of 13 patients are alive in CR with a median follow-up of 30 months [22]. In our study, there was no uniform approach for chemotherapy post relapse. 40% of patients received ICE as second-line therapy for relapse while 36.7% received CCE. CR was reached in 66% of patients that received ICE while 36.3 % achieved CR in the patients that received CCE.

**Table 4:** Recurrent Wilms’ tumor: clinical evidence and studies reports

Study (year)	Group	Patients	Chemotherapy	ASCT	EFS (%)	OS (%)	Ref.
Abu Gosh et al. (2002)	CCG	11	ICE	No (2)	3-year (63.6)	63	[21]
Campbell et al. (2004)	Chicago	13	7/13 cyclophosphamide–etoposide–carboplatin	No (1 or 2)	4-year (60)	73	[22]
Malogolowkin et al. (2007)	NWTS-5	60	Cyclophosphamide–etoposide carboplatin–etoposide	No	4-year (42.3)	48	[23]
Sprea co et al. (2008)	AIEOP	20	ICE	MEC (8/15)	3-year, 56	55	[24]
Hale et al. (2008)	UK CCLG	45	Cyclophosphamide–etoposide carboplatin–etoposide	Melphalan		66	[25]

*AIEOP* Associazione Italiana Ematologia Oncologia Pediatrica, *CCG* Children’s Cancer Group, *CCLG* Children’s Cancer and Leukemia Group, *ASCR* autologous stem cell rescue; *EFS* event-free survival, *ICE* ifosfamide–carboplatin–etoposide, *MEC*: melphalan–etoposide–carboplatin, *NWTS* National Wilms Tumor Study, *OS* overall survival

Our study had limitations, considering the small number of the study group as well as the absence of the risk-oriented approach for the treatment of relapse from the start.

## Conclusions

Radiotherapy post-relapse played a major role in improving the outcome of relapse. The state of lymph node positivity should be taken into consideration when treating relapsed local stage III cases as they proved to have a worse outcome than other relapsed stage III patients. Risk tailored therapy is recommended for a standardized therapeutic approach at our institution that is stratified according to individualized risk factors.

## Supplementary information

**Additional file 1: Table S1.** Relapsing Wilms Tumor Regimens Treatment Protocols.

## Data Availability

The datasets used and analyzed during the current study are available from the corresponding author on reasonable request.

## Abbreviations

COG: Children Oncology Group
CR: complete remission
FH: favorable histology
HDC: high-dose chemotherapy
LOH: loss of heterozygosity
NWTS: National Wilms Tumor Study Group
PD: progressive disease
RTH: radiotherapy
SIOP: International Society of Pediatric Oncology
WT: Wilms’ tumor
