# Supplementary material for: Relapsed Wilms’ tumor in pediatric patients: challenges in low- to middle-income countries—a single-center experience
Source: J Egypt Natl Canc Inst. 2020 May 1;32:21. doi: 10.1186/s43046-020-00032-6 (PMC13317090; doi:10.1186/s43046-020-00032-6)
Supplement: Supplementary file 1 — Additional file 1: Table S1. Relapsing Wilms Tumor Regimens Treatment Protocols. [file 43046_2020_32_MOESM1_ESM.docx]

**Table S1: Relapsing Wilms Tumor Regimens Treatment Protocols**

**ICE regimen for high-risk Recurrent Wilms Tumor**

| **Day** |  | **1** | **2** | **3** | **4** | **5** |  |
| --- | --- | --- | --- | --- | --- | --- | --- |
| **Carboplatin** | **450mg/m^2^** | **↓** |  |  |  |  |  |
| **Etoposide** | **100mg/m^2^** | **↓** | **↓** | **↓** | **↓** | **↓** |  |
| **Ifosphamide** | **1800mg/m^2^** | **↓** | **↓** | **↓** |  |  |  |
| **Mesna** | **1800mg/m^2^** | **↓** | **↓** | **↓** |  |  |  |

In CCE carboplatin is given on 200mg/m^2^ and Cyclophosphamide replaces Ifosphamide at a dose of 1000mg/m^2^. Mesna is given on 350mg/m2 at hour 0, 4and 8 from cyclophosphamide.

CCE is given on 3 days

**Relapsing protocol**

| week | 1 | 4 | 7 | 10 | 13 | 16 | 19 | 22 | 25 |
| --- | --- | --- | --- | --- | --- | --- | --- | --- | --- |
|  | P | P | C | P | P | C | P | P | C |
|  | E | E |  | E | E |  | E | E |  |

P: Carboplatin 500mg/m^2^ x 2 d IV

E: Etoposide 100mg/m^2^ x 3d IV
